# Level of heavy metals and environmental pollution index in Ahvaz, Southwest Iran

**DOI:** 10.1038/s41598-024-64192-4

**Published:** 2024-06-26

**Authors:** Sara Mansouri Moghadam, Khoshnaz Payandeh, Azita Koushafar, Mohioddin Goosheh, Maryam Mohammadi Rouzbahani

**Affiliations:** 1 Department of Environment, Ahvaz Branch, Islamic Azad University, Ahvaz, Iran; 2 Department of Soil Science, Ahvaz Branch, Islamic Azad University, Ahvaz, Iran; 3 Soil and Water Research Department, Khuzestan Agricultural Research and Training Center and Natural Resources, Agricultural Research, Education and Extension Organization, Ahvaz, Iran

**Keywords:** Ahvaz city, Environmental pollution index, Heavy metals, Human health, HMs zoning, Environmental sciences, Environmental social sciences, Solid Earth sciences

## Abstract

This study was designed to evaluate the concentration of heavy metals (HMs) in the north of Ahvaz, southwest Iran. The soil samples were collected from the agricultural farm and riverside in Karun, for the investigation of the environmental impacts of the selected HMs in the soil of the Weiss and Arab Assad regions. For soil sampling in a period, nine farms were selected from each region, and 10 samples were taken from each agricultural farm. Zoning was done using GIS. The highest of Contamination Factor, Enrichment factor and geo-accumulation index of HMs for Cd (7.84, 73.92 and 2.38), and the lowest value of this index for Cr (0.21, 1.98 and − 2.82), respectively. Furthermore of the farm soil showed that the most toxic effect is related to Cd. The HMs contamination indices of the soil samples showed that the studied HMs had contaminated the agricultural fields. Moreover, the zoning maps of the Co, Cu, Pb and Cr showed that they had not contaminated the soil of wheat fields, but Cd and Zn revealed high contamination levels. The zoning of Ni concentration distribution showed that this metal contamination came from both anthropogenic aspects and geological activities in the region. According to our findings, the EF illustrated high levels of pollution for Cd, Cu, Cr, Pb, Ni, Fe, Mn, Co, and Zn, which seems to be in accordance with the accumulation of agricultural fertilizers (phosphate and nitrate), industrial and human activities in the region.

## Introduction

Type of pollutions in the environment can affect human activities and the health of living organisms^1–3^. Agricultural products are one of the sources of pollution affecting human health^4^, the type and methods of agriculture can be affected by heavy metals (HM_S_) and the entry of these pollutants into the food chain becomes dangerous for human health^5^. HM_S_ are toxic and dangerous pollutants that are called a group of toxic HMs with high density and include some metals such as mercury, lead, cadmium, nickel, copper, zinc, chromium, iron, cobalt, manganese and vanadium^6,7^. HM_S_ exists in various sources of water, soil and air^8^, and the man-made sources of these pollutants include industrial and agricultural activities, transportation and means of transport, sewage and urban waste^9^. Also, volcanoes, erosion and dust storms are the geological factors of HM_S_ entering the environment^10^. Soil pollution is important and dangerous in relation to HM_S_^11^, because as a result of agricultural activities and various industries, they easily enter the soil environment^12^, and these pollutants can enter water and air sources through the soil and finally affect the activities affect the biology of living organisms and humans^13^.

HM_S_ is very perdurable, non-degradable and complex pollutants that can slowly accumulate in various organs such as muscles, bones, liver and kidney^9^. HM_S_ causes loss of appetite, nausea, general weakness of the body and muscles^7^. Also, these pollutants cause cardiovascular diseases, Alzheimer’s, Parkinson’s, neurological and metabolic disorders, and ultimately mutations in the genetic system and DNA and human mortality^14^. The increase in the use of chemical fertilizers, herbicides and pesticides, runoff and wastewater from agriculture has caused the pollution of agricultural soils and has increased the concentration of HM_S_ in surface soils^15,16^. One of the most important actions that reduce the introduction of dangerous substances, especially HM_S_, into the food cycle and the human body is soil stability and soil protection^17^.

The existence of underground oil and gas reserves, the plains and fertile lands, and the water rich rivers are among the characteristics of this region. The location of oil, gas, petrochemical, steel, pipeline, carbon block, sugarcane industries, large metallic and nonmetallic industries, the existence of large power plants, giant dams, a rapid growth rate of the population of this region, the existence of many vehicles, urban traffic, the lack of proper infrastructure for transportation, and lack of a sewage collection network are the most critical issues that threaten the health of humans, environment and other living things in this area^8,18^. Due to the high potential of Ahvaz and Shushtar for the production of various products, strategic and industrial crops and the impact of industrial activities such as refineries, petrochemicals, oil, gas, steel industries, sugarcane factories and oil and gas companies; improper use of fertilizers, pesticides and unhealthy irrigation, increases the importance of this study. Therefore, the existence of any metal element, especially HM_S_ in the wastewater entering the soils and water source can be considered as a potential pollution and danger agent. Ahvaz and Shushtar, a southwestern metropolis of Iran located in a dry region, have been recognized as two of the most polluted cities in Asia and the world in recent decades due to severe climate change and dust storms^18^.

A large area of the studied areas of Arab Asad and Weis in the north of Ahvaz city is under the cultivation of agricultural crops, and there is diversity and prosperity of agriculture and the production of many products such as vegetables, grains and summer crops in these fields. Also, agriculture and the production of crops for use in the diet of the people are carried out on a large scale in many Arab Asad and Weis agricultural fields due to the presence of the Karun River on the edge of the agricultural lands and the suitability of the climatic conditions of the study areas, which considering that the direction Cultivation of agricultural crops, use of chemical fertilizers, pesticides and herbicides are common and common, as well as pollutions in important and numerous industries around the studied agricultural lands and the Karun River can lead to the entry of potentially toxic HMs affect the environment of the region, therefore, considering the existence of many industries in Khuzestan province and many studies in the field of increasing pollution in soil and water in this region, it seems that it is necessary to monitor potentially toxic HMs continuously and intermittently.

Therefore, considering the importance and extent of agriculture in the studied area, the importance of toxicity, pathogenicity and carcinogenicity of heavy metals, this research was conducted with the aim of evaluating heavy metals in the soils of agricultural fields in Weis and Arab Asad regions in the north of Ahvaz city, southwest of Iran.

## Materials and methods

### Study area

**Figure 1:**
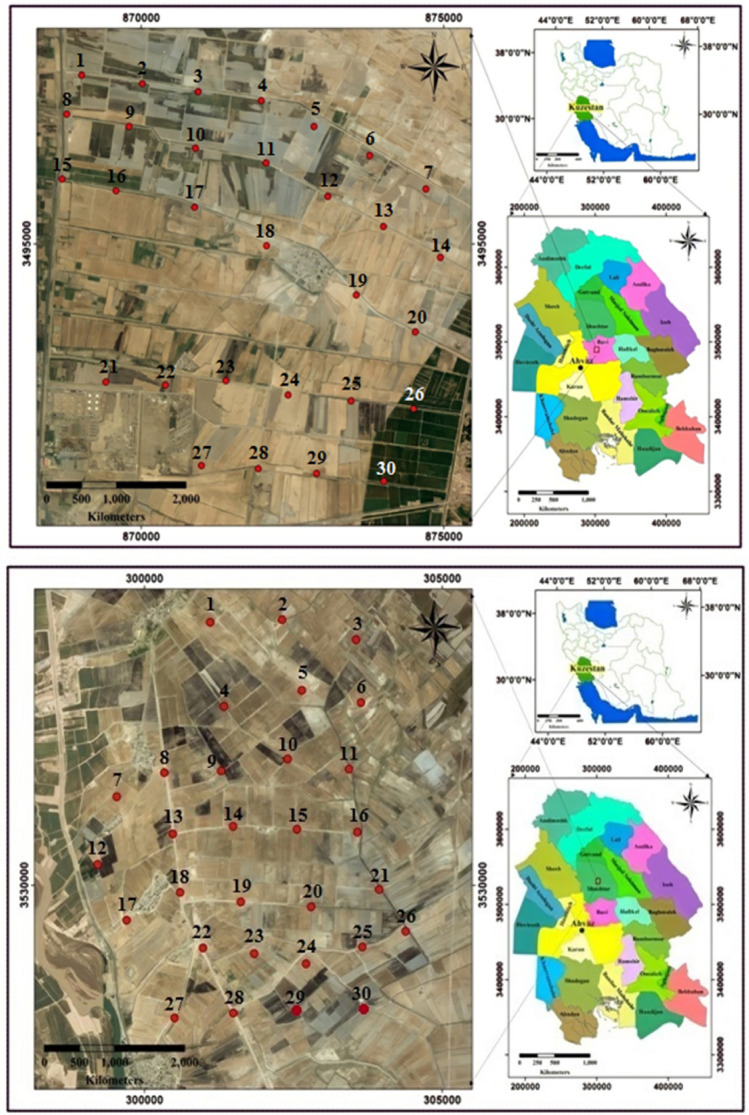
Location map of the sampled site with sampling points ((**a**): Arab Asad region (first part from downstream of Shushtar city to Qir dam; (**b**): Weiss region (second part sampling from Bandaqir to the north of Ahvaz city).

Ahvaz is the capital of the Khuzestan Province, southwest of Iran. Ahvaz, with an area of 185 square kilometers as the center of Khuzestan Province, is located in the southwest of Iran and in the north of the Persian Gulf. It is located 31° 19′ 13″ N, 48° 40′ 9″ E. Ahvaz is one of the largest cities in Iran^19^. The dominant wind direction is northwest to southeast. The population of this province is estimated to be 1.3 million people. It borders Iraq on the west and the Persian Gulf on the south^20^. According to the research objectives, this area was first divided into two parts: the first part, including agricultural lands downstream of Shushtar city, begins and ends at the bitumen dam. These lands are characterized by having medium to semi-heavy textured soil with relatively good drainage and a deep-water table in summer and semi-deep to shallow in autumn and winter. These lands are located between the Shatit and Gargar rivers. The second part starts from the bitumen dam and covers the north of Ahvaz city. The characteristics of these lands have soil with a medium-to-heavy texture and with moderate-to-poor drainage conditions, with a semi-deep water table in summer and shallow in autumn and winter. The soil of the studied area consists of alkaline and calcareous, so the copper absorption efficiency in this soil is very low. A location map showing the sampled site with sampling points in the agricultural lands downstream of Shushtar and north of Ahvaz is shown in Fig. 1. According to the characteristics of the soil of this region based on the standard classification (based on the USDA soil taxonomy), it was determined that the soil of this region consists of entisols and aridsols types^21^.

### Soil sampling

**Figure 2:**
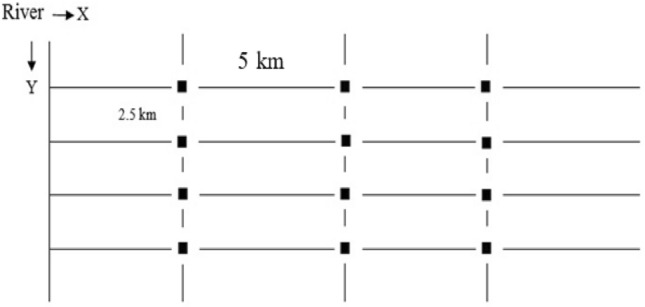
Hypothetical geographical network with three lengths and four latitudes for sampling in each of the two study areas (the intersection of the two axes X and Y are the sampling areas, and the numbers above each location are the sampling location code).

In this study, soil sampling and the measurement of HMs were performed in the surface soil of the Arab Asad region and, in some parts, from downstream of Shushtar city to the place of Qir dam using standard methods^22^. The selected soil sampling in this region was performed according to the soil quality and geographical coverage. Soil Samples were taken based on pollutant inputs, sources of pollutants, the agricultural farms, and the direction of the river in Ahvaz, southwest, Iran. A network with a dimension of 6 km (in terms of longitude) at 5 km along the river was selected to collect soil samples in each of the dual regions. In the longitude direction (perpendicular to the river flow), by taking a distance from the river, three soil samples were taken at a distance of 2.5 km from each other (with a vertical distance from it). In the latitude direction (along the river route), three axes with a distance of 5 km each (a total of three points in each latitude) soil sampling was performed. In this way, a hypothetical geographical network was created with three lengths and four latitudes (12 points or intersections for sampling) (Fig. 2). The spatial variations of HMs were measured to an average depth of 0–30 cm. For soil sampling in a period, nine farms were selected from each region, and 10 samples were taken from each agricultural farm.

### Analyses of samples

The soil samples were collected from each of the two study areas and kept in polyethylene bags. Then, the particles smaller than 63 mm in diameter were separated and immediately transferred to a laboratory for the characterization of their physical and chemical properties. Soil texture (percentage of sand, silt, and clay), and pH levels were determined by the hydrometric method and pH meter. After that the soil samples were treated with a few drops of hydrochloric acid and 7 mL of hydrofluoric acid before being heated in a water bath at 100 °C until nearly dry. Then, they were ground using a mill and weighed one gram of each with a digital scale and were ashed for 5 h at 550 °C. After cooling the samples, 7 ml of 65% HNO_3_ and 35% HCl were added and gently closed the cap to enable the sample to remain overnight for reactions to take place. Next, it was heated in a water bath to dry. After hydrochloric acid (HCl) and nitric acid (HNO_3_) were added to soil samples in a 3:1 ratio (HNO_3_:HCl^23^. Then, the dissolved samples were passed through a paper strainer and were volumized in a 20 ml volumetric flask.

In this study used Inductively Coupled Plasma Optical Emission Spectroscopy (ICP; model Varian 710-ES) for detection and measurement of HMs^24^. We dilute the 1 ml of diluted solution with 9 ml of deionized water. In accordance with standard procedures and protocols, the ICP-OES was used to measure the amount of HMs^1^. Dehumidified samples were ground in a rustproof steel grinder (< 0.25 mm), and the total intents of the above-mentioned HMs were determined using ICP-OES. Samples were analyzed in triplicate for certifying the measurement precision and accuracy in the entire analytical procedure. Synchronously, blank reagent determinations were used to rectify the appliance readings. The obtained results of average recoveries from certified reference material analysis and the determined limit of detection (LOD) and recovery (%) for the measured HMs were a range 0.001–0.015 mg kg^−1^ and 93.75–109.9%. After evaluating 20 samples using standard calibration solutions, the ICP-MS was tuned and calibrated. The number of HMs in each sample was determined to the extent of ppb detection. The samples were analyzed in triplicate for certify the measurement precision and accuracy of the entire analytical procedure. In addition, 90 sediment samples from the soil of the studied region were separately sent to the laboratory to measure Pb isotopic ratios. The collected samples were analyzed in the advanced analysis laboratory of the University of Ahvaz. Quality assurance (QA) and quality control (QC) were assessed by measuring reference material blanks and the NIST 2710 standard. This provided an accuracy of 100 ± 5% (n = 15) while the accuracy of duplicate samples was 6–4%. To ensure the data quality of the samples, the standard reference material (SRM) 2710 was applied simultaneously to 15% of the soil collected from the surface soil samples.

### Contamination factor (CF)

For the assessment of the contamination of toxic metals, the CF was applied (Eq. 1)^8,25^.

1 $${\text{CF }} = {\text{ C}}_{{\text{o}}} \div {\text{ C}}_{{\text{n}}}$$

Which Cn is the concentration of each element in the soil, and Co is the average concentration of each element in the natural background^8,26^. According to the classification provided by Huckanson, CF < 1 demonstrate low, 3 > CF ≥ 1 moderate pollution, 6 > CF ≥ 3 high pollutions, and 6 ≤ CF severe pollution^26^.

### Pollination index (PI)

The PI was calculated for checking the quality of the experimental soil for each HMs^27^.

2 $${\text{PI}} = {\text{ C}}_{{\text{n}}} /{\text{B}}_{{\text{n}}}$$

where C_n_ refers to the measured concentration in the sample, and B_n_ is the measured concentration of the HM_S_ in the Earth’s crust (Table 1)^27^. The pollution index was classified as follows: This index was classified into three levels, including; PI ≤ 1 is equivalent to low pollution level, 1 < PI ≤ 3 is equivalent to a moderate pollution level, and PI > 3 is equivalent to a high pollution level.

**Table 1:** Maximum and minimum values of the PI and IPI in the samples.

Index	Type	As	Cd	Cu	Cr	Ni	Pb	V	Zn
PI (mg.kg^−1^)	Min	0.021	0.04	0.17	0.23	0.64	0.69	0.0013	0.35
	Max	1.23	3.67	1.9	2.2	2.4	8.52	1.5	4.82
IPI	Mean	0.91	2.01	1.5	1.9	2.3	2.04	1	2.92

### Integrated pollution index (IPI)

The IPI is the average value of the PI of each element^28^. This index is classified as the low amount of element IPI ≤ 1, medium amount of element IPI ≤ 2, and high amount of element IPI > 2 = High levels of pollution^28^. In many indicators of HMs pollution in the soil, a reference value is used and the severity of pollution is determined for a specific metal, but in the IPI index, the total HMS pollution in the studied soil is assessed cumulatively (Table 1).

### Nemro integrated pollution index (NIPI)

The advantage of NIPI over other indicators is that in this index, the contamination risk for all metals studied in the region is determined^8,29^. This index was classified into five levels including NIPI < 0.7 without pollution, 1 > NIPI ≥ 0.7 pollution alarm risk, 2 > NIPI ≥ 1 low pollution level, 3 > NIPI ≥ 2 medium pollution level, and NIPI > 3 levels of high pollution, calculated based on Eq. (3)^8,29^:

3 $${\text{NIPI }} = \sqrt {\frac{{{\text{PI}}_{{\text{i}}}^{2} {\text{max}} + {\text{PI}}_{{\text{i}}}^{2} {\text{ave}}}}{2}}$$

### Enrichment factor (EF)

The EF for each metal was calculated from the ratio between the normalizing element and the background value of the HMs, according to the following equation. The measured HMs, according to the sample reference HM, is stable in the Earth’s crust for an estimated EF index^30^. This index is obtained by Eq. (4):

4 $${\text{EF }} = \, (Cx_{{{\text{Metal}}}} /{\text{ Fe}})_{{{\text{Sample}}}} \div \, (Cref_{{{\text{Metal}}}} /{\text{ Fe}})_{{{\text{Background}}}}$$

where C_ref_ is the concentration of the reference element in the sample, and C_x_ is the concentration of the element considered in the sample. A geological origin is the most essential factor in determining the reference element. The reference element in determining the enrich HM_S_ sent factor is an element with a purely geological origin. In this research, the iron (Fe) element was used as a reference HM_S_. According to the classification, EF < 2 low pollution, 5 > EF ≥ 2 moderate pollution, 20 > EF ≥ 5 high pollution, 40 > EF ≥ 20 very high pollution and EF ≤ 40 indicate extremely high pollution^30^.

### Geo-accumulation index (Igeo)

The Igeo can determine the degree of soil pollution. The Igeo is a common method for assessing soil pollution by HMs. Therefore, the Igeo can be used to determine the severity of the pollution^31^. This index is estimated by Eq. (5) ^25^:

5 $${\text{Igeo }} = {\text{Log}}_{{2}} \left( {{\text{Cn}}/{1}.{5} \times {\text{Bn}}} \right)$$

In this regard, Igeo is the geo-accumulation index, Log_2_ is the logarit HM_S_ sic base 2, Cn is the concentration of HMs in the soil, and Bn is the concentration of the field background (average shale). To correct the effects of soil parent materials and natural fluctuations in the content of the given material in the environment and the very little change caused by human activities, a coefficient of 1.5 was used. Seven classes of pollution, Igeo < 0 unpolluted, 0–1 unpolluted to slightly polluted, 1–2 slightly polluted, 2–3 slightly polluted to very polluted, 3–4 very polluted, 4–5 very polluted to heavily polluted and 5 < Ige It is classified as heavily contaminated^31^.

### Statistical analysis

Excel and SPSS were used for the analyses. Sampling and data collection were done by the researcher. Data analysis was used to produce descriptive statistics for the soil pollution indexes. The concentration of HMs was analyzed using SPSS version 25 and Sigma Plot statistical software. Mean data were used to compare significant differences with 95% confidence limits (*P* = 0.05) using the t-test and one-way analysis of variance (one-way ANOVA). The normality of the data was checked using a Kolmograph-Smirnov test. Excel software was also utilized to draw tables and calculate contamination indices.

### Ethical considerations

The Research Ethics Committee of the Islamic Azad University of Ahvaz Branch approved the study protocol. All research experiments were done in the Islamic Azad University of Ahvaz Branch laboratory. The authors are grateful to the Department of Environment, Ahvaz Branch Islamic Azad University (with code IR.AZA.REC.1399.587) for providing the necessary facilities to perform this research. Considering the fact that the data collection method was observation and there were no human participants in the current study, obtaining informed consent is deemed unnecessary according to regulations.

## Results and discussion

### HMs concentration

The statistical description of the concentration of HMs in the surface soil of Weiss and Arab Assad regions is given in Table 2. In this study, the highest of HMs in the soil of Weiss and Arab Assad regions (9013.545 ± 70.77 and 9208.69 ± 93.63 mg/kg) belonged to Fe, respectively. The concentration of Cd had the lowest amount among HMs in the soil of agricultural fields of Weiss and Arab Assad regions, 1 ± 58.10 and 1 ± 56.08 mg/kg, respectively. The statistical analysis of HMs showed that the level of Cd, Pb and Zn in the soil of the fields of the Weiss region was higher than the level in Arab Assad region (*P* > 0.05), but the amounts of Ni and Fe (*P* < 0.05) and Cr, Cu, Co and Mn obtained in the soils of the fields of the Arab Asad region were higher than in the Weiss region (*P* > 0.05) (Table 2).

**Table 2:** Concentration of HMs (Mean ± SD) in the soil of farms in the Weiss and Arab Asad regions.

HMs	Weiss region	Arab Asad region
Cd	1 ± 58.10^a^	1 ± 56.08^a^
Pb	9 ± 83.96^a^	9 ± 29.79^a^
Ni	42.2 ± 34.27^a^	47.3 ± 19.27^b^
Cr	20.1 ± 90.18^a^	21.1 ± 3.40^a^
Co	8 ± 49.99^a^	9.1 ± 6.42^a^
Zn	28.4 ± 68.67^a^	27.2 ± 77.14^a^
Cu	9 ± 22.68^a^	9 ± 70.72^a^
Fe	9013.545 ± 70.77^a^	9208.69 ± 93.63^b^
Mn	226.16 ± 56.33^a^	228.21 ± 31.84^a^

Different letters (a and b) in each row show a significant difference (*P* < 0.05).

The results of soil evaluation indicated that the average levels of Fe and Cd were the highest and lowest in the Weiss and Arab Assad regions (north of Ahvaz). Fe is the principal constituent of the Earth’s outer and inner nuclei and is the sixth most common element on Earth^33^. The results of the study regarding the high levels of Fe in the soil can be explained by the fact that the probable reason for the high levels of this metal in the studied surface soil is that iron is the most abundant constituent of the earth’s crust. Various researchers have reported a high concentration of Fe in the surface soil of different regions^34^. In general, the previous results showed that the Weiss and Arab Assad regions (north of Ahvaz) and their pollution played crucial roles in changing the soil of the studied area and its natural state. The primary sources of Cd in the environment include fossil fuels, waste disposal in plastic bags, insecticides, and sewage sludge in agriculture^35^. The main source of Cd in Iran is phosphorus fertilizers and industries activities. The soil of farms in the Weiss and Arab Asad regions (north of Ahvaz) was used by the available standards introduced by Iran, China, Canada and the United States of America (USQG) for comparing and investigating the level of HMs (Table 3). In Shenyang, northeast of China, Li et al.^36^ studied the HM_S_ contamination of urban soil in an old industrial city and reported that the concentrations of Pb, Cd, and Cu were beyond the study area^36^. While the high concentrations of these HM_S_, along with Zn and Hg, were the same as the concentrations observed in this study, the main reason for this similarity is industries city around the study area including (large metallic and nonmetallic industries, HMsrochemical, steel, pipeline, gas, oil, sugarcane industries), the existence of many vehicles and human activities. In another study, another research team evaluated the level of HMs in the surrounding soils of the Khuzestan steel industry^37^. In agreement with the findings of this study, they reported an increased number of HMs^37^. Furthermore, another research group investigating contaminated soil samples in northern Serbia showed that emission HMs from distance and proximity to the road, industrial, and agricultural activities can play the most important role in the concentration of pollutants entering soil sources^38^. The findings of this study revealed that pollution caused by human activities, especially agriculture and pollutants entering from industries, are the most important cases of HMs entering the soil and pollution of resources.

**Table 3:** Average concentrations of HMs in the Weiss and Arab Asad regions and different countries.

HM_S_ (mg.kg^−1^)	Weiss region	Arab Asad region	Curst	World soils	ISQG	ChSQG	CSQG	USQG
Cd	1	1	3	1.10	3.9	0.3	22	0.06
Pb	9	9	35	25	80	100	91	10
Ni	42.2	47.3	80	18	155	–	50	40
Cr	20.1	21.1	100	42	165	200	87	100
Co	8	9.1	15	4.70	400	250	360	30
Zn	28.4	27.2	85	62	500	300	600	50
Cu	9	9	55	14	40	100	91	20
Fe	9013.545	9208.69	41,000	47,000	–	–	–	–
Mn	226.16	228.21	–	–	–	–	–	600

Iranian Soil Quality Guidelines (ISQG); China Soil Quality Guidelines (ChSQG); Canadian Soil Quality Guidelines (CSQG); the United States of America Soil Quality Guidelines (USQG) ^32,39^.

### Correlation of HM_S_

We use independent T test to compare the mean concentration of different HMs between two regions (Weis and Arab Asad regions, north of Ahvaz). The t-test was used for compare the concentration of HMs in the soil of two regions, based on this test, the amount of Pb (*P*-value = 0.016) and nickel (*P*-value < 0.001) in the two regions has a significant difference and according to the reported averages, their values are significantly lower in Arab Asad region. Pearson correlation coefficient was used to consider the relationship of concentration of different HMs. The amount of Pearson correlation coefficient and it’s *p*-value are expressed in the text. Also, Data analysis by the Pearson method showed that the amounts of HMSs in the soil of the fields in the Weiss region were not significantly correlated (*P* > 0.05) (Cd–Cu R = 0.024; *P* > 0.05, Zn–Cu R = 0.087; *P* > 0.05, Ni–Mn R = 0.107; *P* > 0.05, Pb–Mn R = 0.064; *P* > 0.05), but significant positive correlations were observed between Zn–Pb (R = 0.796; *P* < 0.05), Fe–Cr (R = 0.763; *P* < 0.05). Also, there were significant negative correlations between Cd–Pb (R = − 0.092; *P* > 0.05), Pb–Ni (R = − 0.367; *P* > 0.05), Zn–Co (R = − 0.174; *P* > 0.05), Cu–Cd (R = − 0.024; *P* > 0.05), Fe–Pb (R = 0.189; *P* > 0.05), Zn–Fe (R = − 0.159; *P* > 0.05), and Zn–Mn (R = − 0.055; *P* > 0.05). The results showed that the amounts of HMs in the soil in the Weiss region were not significantly correlated (*P* > 0.05) (Cd–Cu R = 0.037; *P* > 0.05, Pb–Cu R = 0.221; *P* > 0.05, Fe–Mn R = 0.110; *P* > 0.05), but there were significant positive correlations between Cu–Fe (R = 0.886; *P* < 0.05), Cu–Ni (R = 0.678; *P* < 0.05), Ni–Co (R = 0.636; *P* < 0.05). Moreover, there were significant negative correlations between Cd–Mn (R = − 0.006; *P* > 0.05), Pb–Cr (R = − 0.092; *P* > 0.05), Cu–Pb (R = − 0.410; *P* > 0.05), Cu–Mn (R = − 0.069; *P* > 0.05), Fe–Cd (R = − 0.127; *P* > 0.05), Pb–Fe (R = − 0.291; *P* > 0.05) and Co–Mn (R = − 0.010; *P* > 0.05).

### Zoning of HMs

**Figure 3:**
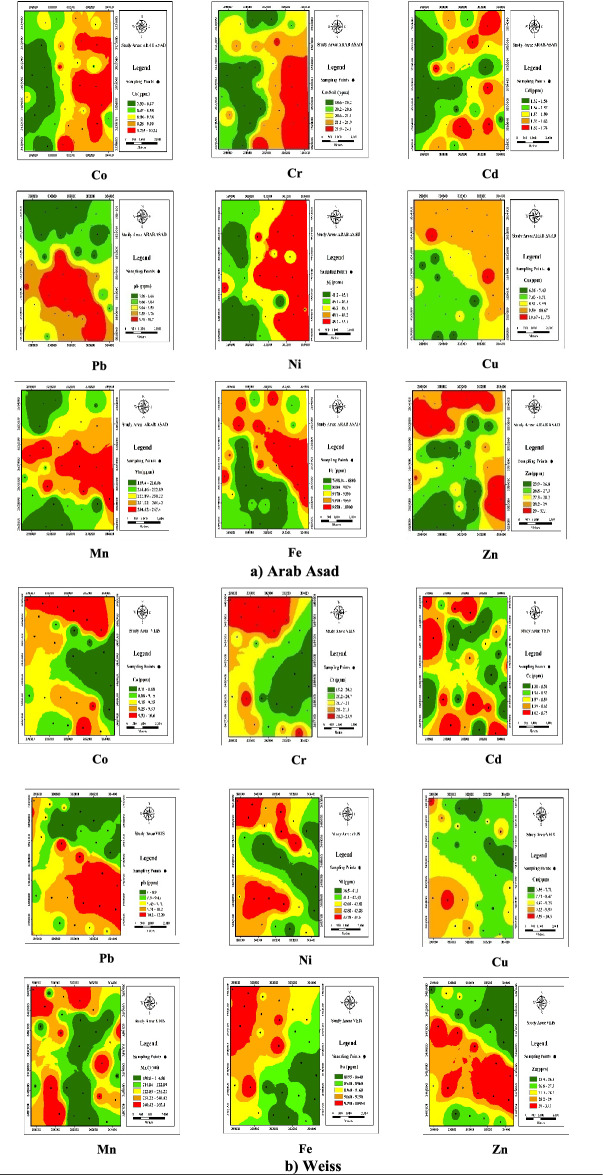
HMs concentration distribution map in surface soils in Weiss and Arab Asad regions (**a**: zoning of HMs Arab Asad; **b**: zoning of HMs Weiss).

Zoning investigated HMs in 180 samples (nine agricultural farms were selected from each region, and 10 samples were taken from each farm) from different zones of the soil of the fields in the Weiss and Arab Assad regions. Zoning was done using GIS. Figure 3 shows the HMs concentration distribution map in surface soils in the Weiss and Arab Asad regions. According to the Iranian National Standard for Human Health (the level of standard is less than 5 mg/kg), the soil cadmium contamination status in the Weiss and Arab Assad regions was classified as non-contaminated (1.38–1.75 and 1.32–1.74 mg/kg, respectively) (Fig. 3). However, according to the level of international standards, including the United States, Japan, Russia, and Canada, the permissible limits are 0.48, 0.4, 0.76, and 1.4 mg/kg, respectively; based on these standards, Cd pollution in the soil was classified in the category ‘soil infected’^40,41^.The Cd contamination in the soil of the studied regions according to international standards showed that farmers in this region had not tested the soil for using chemical fertilizers, especially phosphorus fertilizers. It is worth mentioning that the most important source of the Cd contamination of the soil is the non-optimal consumption of chemical fertilizers (especially superphosphate). Unfortunately, most farmers use this fertilizer every year on their fields where they grow wheat or summer crops. This is a major reason why the soil is contaminated by Cd.

The soil zoning map in the Weiss and Arab Asad regions in terms of chromium element showed that the concentration of this element (19.23–23.9 and 18.6–24.1 mg/kg, respectively) was within the permissible range according to the standards of Iran and Canada, with a permissible limit of 165 and 87 mg/kg, and hence not considered polluted (Fig. 3).In the past decade, soil and groundwater pollution due to the use of chromium in various artificial activities has become a severe concern for plant and animal health^42^. According to the results of many environmental studies, high levels of chromium have been reported in areas near landfills, chromate industries, and highways^43^.

The study of the soil zoning map in the Weiss and Arab Asad regions in terms of cobalt showed that the concentration level of this element (10.6–11.8 and 7.5–10.25 mg/kg, respectively)was within the permissible range based on the standards of Iran and the United States of America (40 and 20 mg/kg, respectively)^41^. It is within the permissible range and thus not considered polluted (Fig. 3). Cu is an essential element of the bodies of living organisms and is widely found in the environment^44^. The human resources of Cu include chemical industries, mineral fertilizers, industrial and mineral effluents and wastes; its natural resources are the minerals and geology of the native soil of the region^45^. The distribution of Cu in the soil depends on human activities, the climate, and the geology of the region. In general, it has less influence in mountainous areas and heights that do not have dense vegetation, so these areas have a lower concentration of Cu in the soil than in the plain areas^46^. The study area in the present study is located in the Khuzestan plain, an area with plain conditions. The evaluation of Fig. 3 showed that the level of Co revealed that the concentration of Cu was (6.95–10.5 and 6.35–11.75 mg/kg, respectively), which according to the standards of Iran, the United States, and Canada with permissible limits of 400, 30, and 360 mg/kg, respectively^40^, this amount was within the allowable range and hence not considered contaminated (Fig. 3). Cu is one of the essential HMs in the bodies of living organisms that is widely distributed in the environment^47^. Copper metal and alloy are used in electric wire and rod, various metal alloys such as silver, jewelry, in computer hardware, coins, thermometers, and thermocouples^48^. The distribution of nickel concentration in the soil of the Weiss and Arab Asad regions shows that the concentration of Ni was (36.5–45.6 and 41.2–53.1 mg/kg, respectively).According to Iranian standards, the soil was not contaminated (155 mg/kg), but according to the standards of America (40 mg/kg) and Canada (50 mg/kg), it was contaminated (69) (Fig. 3). By interpreting zoning maps, it can be inferred that the source of Ni contamination is two significant cases: materials from parent rocks and soils and human activity. In terms of the origin of the parent material, the distribution of Ni is usually uniform in the depth of the soil profilebut is higher on the soil surface, so that it has the highest accumulation at higher levels^49^. In terms of human origin, it also depends on two factors: agricultural and industrial activity^49,50^. The results of this study showed that Ni was distributed in the soil of the region; it seems that in the case of the Arab Asad region, the source of nickel contamination is the native material. The investigation of the soil zoning map of the Weiss and Arab Asad regions in terms of lead element demonstrated that the concentration of Pb (0–12.2 and 7.96–10.7 mg/kg, respectively) in terms of Iranian and Canadian standards, with allowable limits of 80 and 91 mg/kg, respectively, was within the allowed range and thus not considered polluted^51^ (Fig. 3).

Pb is naturally present in all environments and ecosystems. However, most of the lead concentrations found in the environment results from anthropogenic activity. The increase in lead in the environment from human activities stems from the burning of fossil fuels, mining activities, and industry^52^. This metal is a powerful toxin and is harmful even in minimal amounts^17^. Due to the known toxicity of Pb and its damage and effects on humans and other living organisms, it is one of the most critical environmental pollutants^17^. The most important and significant sources of pollutants related to Pb are industries and vehicles^35^. Industries such as plating, battery manufacturing, and the production of electronic components are among the most pollutant industries^53^.

The concentration of zinc according to the zoning maps was 20.25–37.70 mg/kg in the Weiss region and 23.90–33.10 mg/kg in the Arab Asad region (Fig. 3). In this study, the distribution of zinc in the studied regions was within the permissible range (500 mg/kg) according to the standards of Iran and Canada, but according to US standards with a level of 50 mg/kg, it can be considered contaminated^40,41^.

Regarding the zoning of the distribution of Fe and Mn, the critical levels of Fe and Mn are 8 and 6 mg/kg^54^. The result of this study regarding the concentration of Fe and Mn in the soil of the Weiss and Arab Asad regions showed that the soil was severely contaminated (Fig. 3).The findings showed that the most important source of contamination for these two HMs in the soil was the use of iron and manganese sulfate fertilizers and organic fertilizers. According to interviews with farmers, it was found that these fertilizers are used during the summer growing season, which farmers say is without soil testing and is often used much more than recommended. Due to the high intensity of Fe and Mn contamination in the soil of agricultural fields in the Weiss and Arab Assad regions, causing disturbance and contamination in the soil, it can be concluded that many divalent metal cations such as manganese, iron, cobalt, nickel, copper, and zinc are structurally very similar and replaced by each other.

### Evaluation of the environmental index

The CF of Cd, Ni, Co, and Mn in the soils of agricultural fields in the Arab Assad region was higher than in the Weiss region, but it was lower in the Arab Assad region for Pb, Zn, and Cu. The highest of CF to Cd (7.84) and the lowest value of this index to Cr (0.21). The pattern of HMs CF values in the soil of wheat fields of the Weiss and Arab Assad regions was in the form of Cd > Mn > Ni > Zn > Cu > Pb > Co > Cr. The enrich HM_S_ sent factor of Cd, Pb, Cr, Zn, Cu, and Mn was also higher in the soil of Weiss than in the soil of Arab Assad, and the EF of Ni and Co in the soil of the Weiss was lower than in Arab Assad. The highest amount of EF was related to Cd (73.92), and the lowest value of this index was related to Cr (1.98). The pattern of EF values in the soils of wheat fields of Weiss and Arab Assad regions was in the form of Cd > Mn > Zn > Cu > Pb > Ni > Co > Cr. The Igeo index of HMs of Ni, Cr, Co, and Mn in the soil of the Assad Arab region was higher than in Weiss, but the geo-accumulation index for Pb, Ni, and Co was higher in the soil of Weiss region than in the of Arab Assad region. The highest value of the HMs Igeo index was related to Cd (2.38), and the lowest value of the earth geo-accumulation index was related to Cr (− 2.82). The pattern of geo-accumulation index values of HMs in the soil of the Weiss and Arab Assad regions for cadmium chrome was in the form of Cd > Mn > Ni > Zn > Cu > Pb > Co > Cr. Regarding HMs in soils, the values of environmental indicators, including the PI and NIPI, in the Arab Asad region were higher than in the Weiss region (Table 4). Cd is one of the HMs that has no role and activity in the body of living organisms and if it enters the body of animals and humans, it causes toxicity and causes disease and carcinogenesis in the long term^15^. In the past years and recent researches and studies, researchers conducted many studies on the evaluation of Cd at different levels of the environment, which reports indicated the contamination of HMs. In other researches, contamination of Cd has been reported on rice and wheat agricultural products in the agricultural fields of Khuzestan province and around the cities of Ahvaz and Bavi^8,22,32,37,55^. Forest fires and volcanoes are important sources of Cd entry into the environment, but the main sources of cadmium metal entry are through anthropogenic activities and industrial waste production^15^. This metal is used in the industry as an anti-friction material, catalyst, anti-rust, compound of alloys. Cadmium is also used in rod protection semiconductors in nuclear reactors, metal plating, ceramic manufacturing, PVC factories and plastic industries, battery production, fungicide compounds, motor oil, rubber manufacturing and photography^17,35^. The main use of this element is as a stabilizer and pigment in plastic and electrolysis industries, but its main part is used in soldering and as an alloy in nickel–cadmium batteries. Phosphate fertilizers are also emitters of cadmium metal in agricultural lands^34^.

**Table 4:** Results of environmental index in agricultural soils of Weiss and Arab Assad regions.

Regions	Metals (mg.kg^−1^)	CF	EF	Igeo index	PI	NIPI
Weiss	Cd	7.83	73.92	2.38	1.02	5.67
	Pb	0.58	5.46	− 1.38		
	Ni	2.11	4.06	0.49		
	Cr	0.21	2.01	− 2.82		
	Co	0.26	2.41	− 2.55		
	Zn	1.03	9.72	− 0.54		
	Cu	0.92	8.72	− 0.70		
	Mn	3.74	35.29	1.32		
Arab Assad	Cd	7.84	72.59	2.38	1.95	5.69
	Pb	0.55	5.07	− 1.46		
	Ni	2.36	4.45	0.65		
	Cr	0.21	1.98	− 2.81		
	Co	0.27	2.50	− 2.48		
	Zn	0.99	9.15	− 0.60		
	Cu	0.90	8.31	− 0.74		
	Mn	3.81	35.19	1.34		

Due to the growing population, the expansion of urbanization, industrialization, oil and gas extraction, production of steel mills, cultivating more land for food supply, more use of agricultural fertilizers, wastewater, and the existence of many vehicles, urban traffic in Ahvaz in the past decades, this region is heavily affected by a large volume of environmental pollutants. In the same study conducted by Rastmanesh et al.^55^ in Abadan, Iran, on the impact of Abadan Petrochemical complex and petroleum refinery on heavy soil metals, it was found that the main sources of HMs production was agricultural fertilizers, industries (oil, gas, petrochemical) and vehicles (high traffic),which is in agreement with the findings of this study. The high values of 208Pb/206Pb and 207Pb/206Pb in the soil of the studied region indicates that the pollutants were of human origins, like industrial activities for the Pb metal. According to the results of the present study, the excessive use of agricultural fertilizers and the location of many industries in this region are among the most important HMs contamination factors in the region. NIPI is calculated for each sample point. The advantage of this index compared to other indices is that in this index, the pollution risk for all the HMs that are studied in the region is determined. The results obtained from Table 4 show the contamination index of all samples ranges in agricultural soils of Weiss (5.67) and Arab Assad (5.69) regions. The average value of NIPI index in studied performed by Halil et al.^56^ was 7.18. The reference element in determining the enrichment factor is an element that has a completely natural origin. Zn, Ti, Fe, Al and Sr are usually used as reference HMs in environmental research^56^. The average EF, Cd and Mn had high levels of contamination and Zn contamination was high. Halil et al.^56^ reported in their study that average EF, Ni, Cu and Pb had high levels of contamination and Zn contamination was high that same with the result of this study. Considering that the EF the increase in the concentration of an element compared to its natural concentration in the soil^4,31^, also in environmental analysis, one of the important factors for evaluating the concentration of elements under the influence of anthropogenic factors and it is natural^22,32^, so it can be concluded that the agricultural soils of Arab Asad and Weis regions are affected by heavy metals from man-made sources in the region. In numerous researches and studies, the entry of heavy metals through anthropogenic means such as industrial and agricultural activities has been reported^1,12,23,28^. Based on the results of study by Kamalu et al. in 2011, the oil extraction process has resulted in the spilling of toxic drilling by-products into local and international soil and water bodies surrounding the study area which agrees with the findings of the current study^57^. In a study on HMs in Huixian wetlands in southern China, Huang et al. reported that there are moderate to high ecological hazards due to HMs in the coastal soils of this wetland^58^. The contamination factor for Pb, Cu, Co, and Cr was less than 1, indicating the low contamination of these metals in the soil. The C_f_ for Se was in the range of 1–3, indicating moderate contamination for this metal^59^. The C_f_ for Cd and Mn at the third station was higher than 3, which showed the high contamination of this element. The contamination factor for HMs implies the concentration of these HMs is higher than that of the earth’s crust and shale. In some countries, such as New Zealand, Finland, and particular areas of China, the available HMs in the soils is naturally low^60^.

## Conclusion

The contamination level of soil was evaluated on the basis of indices, namely contamination factor, pollution index, enrichment factor, geo-accumulation index, and Nemro integrated pollution. Considering that these two regions are located in the plain of Khuzestan and this region is one of the most fertile regions of Iran in terms of agriculture and the planting of wheat and other crops, it is of great importance and plays a strategic role in providing food to the people. So the investigation of HMs in the soil of this Areas and using ways to reduce these harmful substances and enter the food cycle play a very important and effective role in increasing people’s health. This study was to investigate the pollution of HMs (Cd, Cu, Cr, Pb, Ni, Fe, Mn, Co, and Zn) in the soil caused by the activity of agricultural fertilizers, vehicles, and industries in the north of Ahvaz, southwest of Iran. According to our findings, the enrichment factor (EF) illustrated high levels of pollution for Cd, Cu, Cr, Pb, Ni, Fe, Mn, Co, and Zn, which seems to be in accordance with the accumulation of agricultural fertilizers (phosphate and nitrate), industrial, and human activities in the region. The Average level of analyzed HMs in the soil was greater than the average standard levels in soil. According to the results of the enrichment factor, it seems that HMs in the soil was affected by anthropogenic activities. The findings of this study revealed that human activities are the main factor that significantly affects the concentration of HMs. HMs contamination indices in the soil showed that the studied HMs were contaminated in the soil of agricultural fields. According to the zoning maps of Co, Cu, lead, and Cr, there was no contamination in the soil of wheat fields, but Cd and Zn were highly contaminated and also, the Assessment of soil pollution of wheat farms showed that the best effect of toxicity was related to Cd. Moreover, the zoning of nickel-metal concentration distribution showed that the source of this metal contamination had geological activities and anthropogenic aspects in the region. The major limitations this study include the large number of tables and figure in this manuscript, the results related to heavy metals in water and cultivated wheat are not presented and will be published in future articles. It is suggested to investigate the ecological risk assessment of heavy metals in wider areas of agricultural fields in the Karun River catchment area. Also, the assessment of the risk of heavy metals in other agricultural products cultivated in the northern areas of Ahvaz city should also be investigated. On the other hand, the risk assessment of health hazards and ecological hazard of arsenic and mercury metals in agricultural soils and cultivated crops in fields in important areas of Khuzestan province should be investigated.

### Supplementary Information

Supplementary Tables.

## Data Availability

All data generated or analyzed during this study are included in this published article [and its Supplementary Information Tables, Fig. 3a-Arab and Fig. 3b-Weiss].
