# Supplementary material for: Level of heavy metals and environmental pollution index in Ahvaz, Southwest Iran
Source: Sci Rep. 2024 Jun 26;14:14754. doi: 10.1038/s41598-024-64192-4 (PMC11208444; doi:10.1038/s41598-024-64192-4)
Supplement: Supplementary file 1 — Supplementary Tables. [file 41598_2024_64192_MOESM1_ESM.docx]

**Level of Heavy Metals and Assessment Ecological Risk in Ahvaz, Southwest Iran**

**Sara Mansouri Moghadam^1^, Khoshnaz Payandeh*^2^, Azita Koushafar^3^, Mohiaddin Goosheh^2,4^, Maryam Mohammadi Rouzbahani^3^**

**Table S1:** Maximum and minimum values of the pollution index (PI) in the samples

| **Zn** | **V** | **Pb** | **Ni** | **Cr** | **Cu** | **Cd** | **As** | **PI (mg.kg^-1^)** |
| --- | --- | --- | --- | --- | --- | --- | --- | --- |
| 0.35 | 0.0013 | 0.69 | 0.64 | 0.23 | 0.17 | 0.04 | 0.021 | Min |
| 4.82 | 1.5 | 8.52 | 2.4 | 2.2 | 1.9 | 3.67 | 1.23 | Max |

**Table S2:** Integrated pollution index for the studied samples

| **Zn** | **V** | **Ni** | **Pb** | **Cr** | **Cu** | **Cd** | **As** | **Metals (mg.kg^-1^)** |
| --- | --- | --- | --- | --- | --- | --- | --- | --- |
| 2.92 | 1 | 2.04 | 2.3 | 1.9 | 1.5 | 2.01 | 0.91 | (IPI) |

**Table S3:** Enrichment factor in samples

| **Enrichment intensity** | **Enrichment factor** |
| --- | --- |
| No enrichment | EF≤ 1 |
| Low enrichment | 1< EF< 3 |
| Moderate enrichment | 3< EF< 5 |
| Relatively rich enrichment | 5< EF< 10 |
| Severe enrichment | 10< EF< 25 |
| Very severe enrichment | 25< EF< 50 |
| Extremely rich enrichment | EF> 50 |

**Table S4:** The values of the geo-accumulation index used in determining soil pollution

| **Pollution intensity** | **Geo-accumulation index (Igeo)** |
| --- | --- |
| Uncontaminated soils | Igeo ≤ 0 |
| Soil without pollution to moderately contaminated | 0< Igeo < 1 |
| Low/ moderate pollution | 1< Igeo < 2 |
| Moderate to highly contaminated soils | 2< Igeo < 3 |
| High pollution | 3< Igeo < 4 |
| High to extremely contaminated soils | 4< Igeo < 5 |
| Extremely pollution (extremely contaminated soils) | Igeo > 5 |

**Table S5:** the statistical parameters (t-test) of heavy metals in the soil of fields in Weis and Arab Asad regions (north of Ahvaz).

| **Heavy metals (mg.kg^-1^)** | **Region** | **Mean±standard deviation** | **Minimum** | **Maximum** | **P-value** |
| --- | --- | --- | --- | --- | --- |
| **Cd** | Weiss region | 1±58.10 | 1.38 | 1.75 | **461/0** |
|  | Arab Asad region | 1±56.08 | 1.32 | 1.74 |  |
| **Pb** | Weiss region | 9±83.96 | 8.15 | 12.20 | **016/0** |
|  | Arab Asad region | 9±29.79 | 7.96 | 10.70 |  |
| **Ni** | Weiss region | 42.2±34.27 | 36.50 | 45.60 | **001/0 >** |
|  | Arab Asad region | 47.3±19.27 | 41.20 | 53.10 |  |
| **Cr** | Weiss region | 20.1±90.18 | 19.20 | 23.90 | **690/0** |
|  | Arab Asad region | 21.1±3.40 | 18.60 | 24.10 |  |
| Co | Weiss region | 8±49.99 | 6.95 | 10.75 | **113/0** |
|  | Arab Asad region | 9.1±6.42 | 6.35 | 11.75 |  |
| **Zn** | Weiss region | 28.4±68.67 | 20.25 | 37.70 | **333/0** |
|  | Arab Asad region | 27.2±77.14 | 23.90 | 33.10 |  |
| **Cu** | Weiss region | 9±22.68 | 8.11 | 10.60 | **250/0** |
|  | Arab Asad region | 9±70.72 | 7.50 | 10.25 |  |
| **Fe** | Weiss region | 9013.545±70.77 | 8195 | 10950 | **233/0** |
|  | Arab Asad region | 9208.69±93.63 | 7698 | 10900 |  |
| **Mn** | Weiss region | 226.16±56.33 | 202.60 | 285.10 | **726/0** |
|  | Arab Asad region | 228.21±31.84 | 189.60 | 265.40 |  |
